# Activation of Complement Pathways in Kidney Tissue May Mediate Tubulointerstitial Injury in Diabetic Nephropathy

**DOI:** 10.3389/fmed.2022.845679

**Published:** 2022-04-11

**Authors:** Shimin Jiang, Yuanyuan Jiao, Guming Zou, Hongmei Gao, Li Zhuo, Wenge Li

**Affiliations:** ^1^Department of Nephrology, China-Japan Friendship Hospital, Beijing, China; ^2^Peking Union Medical College and Chinese Academy of Medical Sciences, Beijing, China

**Keywords:** diabetic nephropathy, tubulointerstitial injury, complement system, prognosis, WGCNA

## Abstract

**Introduction:**

Key genes involved in tubulointerstitial injury may influence the development and progression of diabetic nephropathy (DN). We investigated whether complement-related genes are linked to the mechanism underlying tubulointerstitial injury in DN.

**Methods:**

We analyzed the microarray data of 17 tubulointerstitial tissue samples from DN patients and 21 normal controls from the Gene Expression Omnibus. A gene co-expression network was constructed, and genes were divided into modules by weighted gene co-expression network analysis (WGCNA). We also investigated the association of C3 and C1q deposits in kidney tissues with a composite outcome of end-stage renal disease or a 50% reduction in the estimated glomerular filtration rate (eGFR) in DN patients. Finally, we performed immunohistochemical analyses of C3, C1q, C5b-9, mannose-binding lectin (MBL), and factor B in kidney tissues.

**Results:**

Nine co-expression modules were constructed using 12,075 genes from the 38 human tubulointerstitial tissue samples. Black module with more genes was positively correlated with tubulointerstitial injury in DN. C3, one of the top 10 genes in tubulointerstitial injury, was verified in an independent dataset; C3 was significantly overexpressed in tubulointerstitial tissue from patients with DN compared to the normal controls. The mRNA level of C3 in renal tubulointerstitium was negatively correlated with eGFR in DN patients (*r* = −0.75; *p* = 0.001). Analysis of the follow-up data of 54 DN patients demonstrated that codeposits of C3 and C1q in kidney tissues were independently associated with the renal outcome in DN (hazard ratio, 2.3, 95% confidence interval, 1.01–5.2, *p* < 0.05). Immunohistochemical analysis showed that patients with higher C1q, C3, C5b-9, MBL, or factor B expression in renal tubulointerstitium were more likely to progress to kidney failure.

**Conclusion:**

Local complement activation of the classical, lectin and alternative pathways appears linked to tubulointerstitial injury and disease progression in DN.

## Introduction

Diabetes is a major health issue worldwide. In 2021, an estimated 537 million individuals had diabetes, and this number is expected to reach 783 million by 2045 (1). Diabetic nephropathy (DN), a microvascular complication of diabetes, is the leading cause of end-stage renal disease (ESRD) in developed countries (2, 3). Despite glycemic control, blood pressure control, and renoprotective agents, the morbidity and mortality of DN are high. Because not all patients with type 1 or 2 diabetes develop DN, there is a need for biomarkers to identify patients who at risk of DN, and for new therapeutic approaches to delay its onset and progression to diabetic ESRD.

The pathologic changes of DN include glomerular and tubulointerstitial lesions (4), both of which contribute to the decline of kidney function seen in DN (5, 6). Because glomerular lesions reflect the course of progressive DN, many studies evaluated glomerular lesions in DN (4, 7, 8). However, few gene expression studies explored the mechanisms underlying tubulointerstitial injury caused by DN. Key genes involved in the tubulointerstitial lesions induced by DN may be predictive of the disease progression and survival of patients with DN. Data mining *via* weighted gene co-expression network analysis (WGCNA) (9) is used to investigate the gene-network signature, co-expression modules, and key genes in diseases. We performed a WGCNA to identify complement-related key genes involved in diabetic tubulointerstitial injury with the aim of elucidating the mechanism underlying DN.

## Materials and Methods

### Data Collection

We downloaded tubulointerstitial transcriptional profiles of DN patients from the Gene Expression Omnibus (GEO) database^1^ on 3 April 2021. GSE104954, which was based on GPL22945 (Human Genome U133 Plus 2.0 Array; Affymetrix, Santa Clara, CA, United States) and GPL24120 (Human Genome U133A Array; Affymetrix) platforms, was used to construct co-expression networks and identify hub genes linked to diabetic tubulointerstitial injury. The microarray dataset enabled transcriptome comparison of the tubulointerstitium of patients with DN (*n* = 17) and control tubulointerstitium tissues from healthy living transplant donors (*n* = 21).

### Construction of Co-expression Network and Module Detection by Weighted Gene Co-expression Network Analysis

We constructed a gene co-expression network using the R package “WGCNA” (9). Clustering is the division of a dataset into several groups according to the similarity between instances; outlier samplers were removed before performing WGCNA. After the GEO expression matrix data were sorted, an appropriate soft-threshold power β was selected and 0.9 was used as the correlation coefficient threshold in the WGCNA. Next, a scale-free topology plot was created using the appropriate soft-threshold power. Finally, a clustering tree and dendrogram plot of co-expression gene modules were generated based on the following major parameters: cutHeight = 0.25, miniSize = 60. The module eigengene (ME), obtained by WGCNA, indicated the expression profile of a given module’s genes (9).

### Identification of Diabetic Nephropathy-Related Modules

Because ME represents the gene expression pattern of a certain module, the correlations between MEs and clinical traits were calculated using Pearson’s test to identify trait-related modules, for both the DN patients and healthy donors. In the module-trait correlation analysis, gene significance (GS) was defined as the *p*-value for each gene in the linear regression between gene expression and the clinical traits. Module membership (MM) was the association between the gene expression value and MEs in a module. A strong correlation between GS and MM in a given module indicates that the genes therein make a marked contribution to both the module and its corresponding clinical traits (10).

### Functional Enrichment Analysis of Diabetic Nephropathy-Related Modules

The modules with strong correlations with DN were selected as DN-related modules for further analyses. The genes therein were mapped to the DAVID dataset^2^ (11), and Gene Ontology (GO) analysis was performed to evaluate their biological functions. A *p*-value < 0.05 after correction was set as the threshold. If there were more than 10 records, the top 10 were extracted.

### Hub Gene Identification

In the module-trait correlation analysis, genes with | MM| > 0.8 and | GS| > 0.4 were considered as those having a significant correlation with a clinical trait. Since the STRING database can integrate data on protein–protein interactions (PPI) (12), a PPI network among the significant genes in a module with a confidence score of 0.9 was created using the STRING database.^3^ Next, the exported data, including source and target node genes, were imported into Cytoscape (version 3.7.1^4^). To identify high-level genes in PPI networks, cytoHubba (a plugin of Cytoscape) (13) was used to calculate the maximal clique centrality (MCC) value of each gene, and the top 10 MCC values in a module were defined as hub genes.

### Gene Expression Omnibus Validation of Hub Gene

Among the hub genes associated with tubulointerstitial injury in DN, complement-related genes were validated in the GSE99325 dataset, which was downloaded from the GEO database. The GSE99325 dataset (based on GPL19109 and GPL19184 platforms) comprised the data of 18 DN patients and 6 controls with tumor nephrectomies. Microdissection into the tubulointerstitial compartment and Affymetrix-based profiling of gene expression in the tubulointerstitium were performed.

### Clinical Validation of Hub Gene

The clinical validation was approved by the Ethics Committee of China-Japan Friendship Hospital (2018-43-K32). Informed consent was obtained from patients for use of their medical data. Patients with pure DN from 2017 to 2019 were analyzed. Renal biopsy specimens were evaluated routinely by light microscopy, direct immunofluorescence microscopy, and transmission electron microscopy. Inclusion criteria were age 18–75 years and an estimated glomerular filtration rate (eGFR) > 15 ml/min/1.73 m^2^ at the time of renal biopsy. Patients with non-diabetic kidney disease were excluded. The follow-up ran from the date of renal biopsy to the day of one of the three endpoints: confirmed ESRD, a 50% decline in the initial eGFR, or the last outpatient visit. ESRD was defined as the need for maintenance dialysis or kidney transplant during follow-up. The length of follow-up was at least 6 months unless a composite outcome (ESRD and 50% reduction in eGFR) occurred before that time. The progressive group included patients with the combined outcome, and the stable group included patients with no definitive renal outcome. The eGFR was calculated using the creatinine-based Chronic Kidney Disease Epidemiology Collaboration equation (14).

For direct immunofluorescence, the intensity of staining of complement C3 and C1q in kidney tissue was semi-quantitatively graded on a scale of 0 ∼ ++++ (−, no fluorescence at low or high magnification; ±, no fluorescence at low magnification, but seemingly visible at high magnification; 1+ or higher, visible at low or high magnification).

### Immunohistochemistry of C1q, C3, Mannose-Binding Lectin, C5b-9, and Factor B

Paraffin-embedded renal tissues were sectioned at 2 μm thickness. After dewaxing and hydration, high-pressure steam repair antigen repair was performed. Endogenous peroxidase was blocked with 3% peroxide-methanol and incubated in goat serum working fluid for 30 min at room temperature. Next, the following primary antibodies (all from Abcam, Cambridge, United Kingdom) were added separately: anti-C1q (ab268120) diluted 1:400, anti-C3 (ab97462) diluted 1:300, anti-C5b-9 (ab55811) diluted 1:500, anti-mannose-binding lectin (MBL) (ab23457) diluted 1:25, and anti-factor B (ab192577) diluted 1:400. After overnight incubation at 4°C, the reactions were visualized by staining with horseradish peroxidase and diaminobenzidine.

Immunohistochemical images were obtained from sections observed microscopically at ×200 magnification (Nikon, Tokyo, Japan) using an integrated digital camera system (Nikon). The average optical density (AOD) was obtained by semiquantitatively analysis of 10 fields in a blinded manner. Image-Pro Plus software 6.0 (Media Cybernetics, Bethesda, MD, United States) was used to analyze the AOD of stained areas.

### Statistical Analysis

Differentially expressed genes between DN patients and normal controls were identified using the ‘‘limma’’ package in R software (version 4.0.5^5^) with the threshold set at | log2-fold change (logFC)| ≥ 1 and an adjusted *p*-value < 0.05. Pearson correlation analysis was performed between tubulointerstitial C3 and eGFR in patients with DN using the Nephroseq v5 online platform.^6^ We compared baseline clinical variables between the stable and progressive groups. Student’s *t*-test and the χ^2^ test were used for analyzing continuous and categorical variables, respectively. A Cox regression analysis was performed to calculate the hazard ratio (HR) and 95% confidence interval (CI) for variables correlated with kidney failure. Univariate Cox regression analysis was conducted to estimate the associations between baseline variables and renal outcome. Variables with *p*-values < 0.1 in univariate analyses were entered into a multivariate Cox regression model. Statistical significance was set at *p* < 0.05.

## Results

### Data Preparation and Evaluation

The expression profiles of 12,075 genes from 38 renal tubulointerstitial samples were subjected to WGCNA. As shown in Supplementary Figure 1, all samples were divided into two clusters: one cluster contained 17 tubulointerstitial tissue samples from DN patients and the other contained 21 samples from the healthy controls.

### Construction of the Weighted Gene Co-expression Network

**FIGURE 1:**
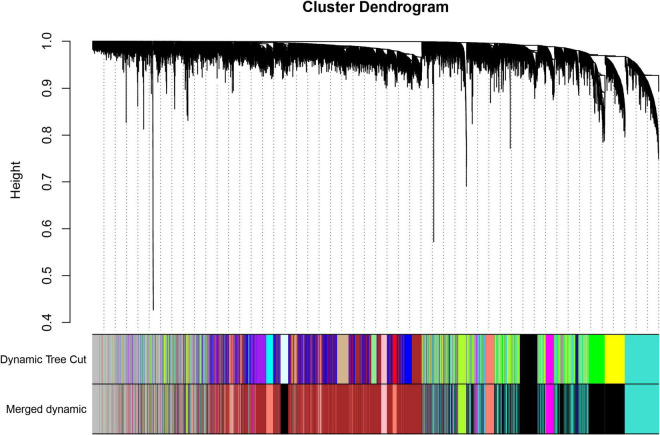
Clustering dendrogram of genes with dissimilarity based on topological overlap, together with assigned module colors.

We selected 11 as the soft-thresholding power to construct a scale-free network when 0.9 was used as the correlation coefficient threshold (Supplementary Figure 2). The co-expression modules, which were confirmed by dynamic tree cutting, were indicated by different colors. WGCNA constructed nine co-expression modules of diverse sizes (Figure 1). The number of genes in the modules ranged from 192 to 3,758 (Table 1).

**TABLE 1:** The number of genes in the nine modules.

Module colors	Number of genes
Black	2,779
Brown	3,758
Greenyellow	303
Gray	1,443
Magenta	353
Midnightblue	192
Pink	387
Salmon	863
Turquoise	1,996

The relationships of 400 selected genes are presented in a network heatmap (Supplementary Figure 3). Genes in the same module were strongly correlated, whereas those in different modules were weakly correlated. Therefore, these modules were independent of other modules.

### Selection of Trait-Related Modules

**FIGURE 2:**
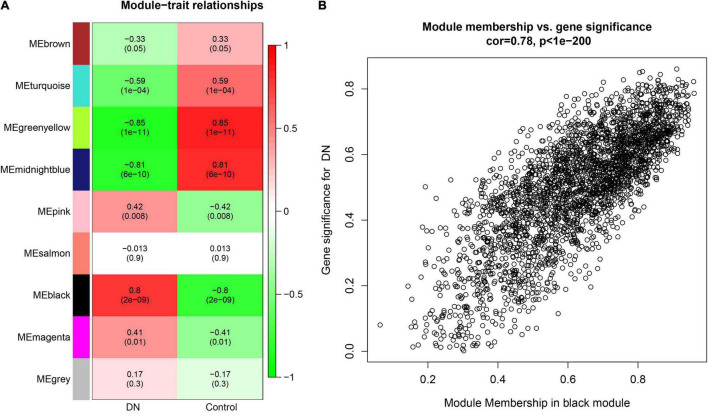
**(A)** Module-trait relationships heatmap. Each row corresponds to a module eigengene (ME), and each column to a clinical trait. The correlation and p values are shown in the cells. **(B)** A scatterplot of gene significance (GS) for diabetic nephropathy (DN) versus module membership (MM) in the black. A highly significant correlation exists between GS and MM in the modules.

The correlations between ME values and clinical traits are shown in Figure 2A. Multiple modules were related to DN. The black module containing 2,779 genes was the most positively related to tubulointerstitial lesions in DN (*r* = 0.8, *p* = 2e−09), whereas the midnightblue (192 genes; *r* = 0.81, *p* = 6e−10), and greenyellow (303 genes; *r* = 0.85, *p* = 1e−11) modules were negatively correlated with tubulointerstitial lesions in DN. Finally, a scatterplot of GS versus MM in the black module was generated (Figure 2B). Most genes were in the upper-right corner, with the GS ranging from 0.4 to 1 and the MM from 0.6 to 1.

### Functional Enrichment Analysis

The top 10 GO terms of the genes in the black module are shown in Table 2. In the cellular components (CC), most were in extracellular exosome, extracellular matrix, membrane, and extracellular space. For biological process (BP), most terms were associated with inflammatory biological processes (e.g., interferon-gamma-mediated signaling pathway and positive regulation of I-kappaB kinase/NF-kappaB signaling), the immune response, and extracellular matrix organization. A molecular function (MF) analysis indicated that these molecules were involved in protein binding. These GO terms should facilitate further research on the role of module genes in tubulointerstitial lesions in DN.

**TABLE 2:** Gene Ontology (GO) enrichment analysis of genes in black module.

Category	TermID	Rank	Count	*p*.adjust	TermName
CC	GO:0070062	1	158	3.79E−20	Extracellular exosome
BP	GO:0060333	2	20	5.30E−11	Interferon-gamma-mediated signaling pathway
MF	GO:0005515	3	308	8.52E−10	Protein binding
CC	GO:0005925	4	37	1.01E−08	Focal adhesion
CC	GO:0031012	5	30	1.30E−07	Extracellular matrix
BP	GO:0006955	6	36	3.73E−06	Immune response
BP	GO:0030198	7	23	1.18E−05	Extracellular matrix organization
CC	GO:0016020	8	100	2.15E−06	Membrane
CC	GO:0005615	9	70	3.07E−06	Extracellular space
BP	GO:0043123	10	20	3.83E−05	Positive regulation of I-kappaB kinase/NF-kappaB signaling

*CC, cellular component; BP, biological process; MF, molecular function.*

### Hub Genes Linked to Tubulointerstitial Injury in Diabetic Nephropathy

Based on the cut-off criteria (| MM| > 0.8 and | GS| > 0.4), 414 genes with high connectivity in the black module were imported into the STRING database to construct a PPI network. A total of 411 nodes and 808 interaction pairs with the highest confidence (0.9) were identified and imported into Cytoscape for visualization. The MCC value of each gene was calculated using the CytoHubba plugin and the top 10 genes in terms of MCC values were screened (Supplementary Figure 4). These key genes, which were centrally located in the network, comprised ADAM metallopeptidase domain 10 (ADAM10), cytoskeleton associated protein 4 (CKAP4, also known as P63), complement C3 (C3), fibrillin 1 (FBN1), tenascin C (TNC), secreted phosphoprotein 1 (SPP1, also known as osteopontin), laminin subunit gamma 1 (LAMC1), TIMP metallopeptidase inhibitor 1 (TIMP1), versican (VCAN), and heat shock protein 90 β family member 1 (HSP90B1).

### Gene Expression Omnibus Validation of C3

**FIGURE 3:**
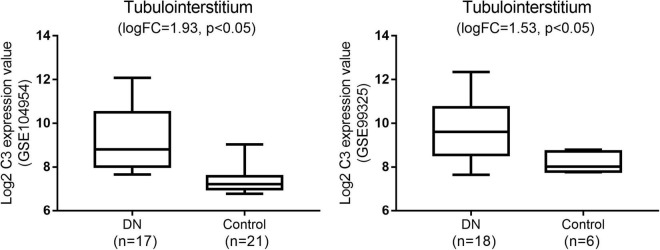
Gene expression omnibus validation: the expression of C3 in diabetic nephropathy (DN) when compared with normal tissues in the GSE104954 and GSE99325 datasets.

Among the hub genes related to tubulointerstitial injury in DN, we further analyzed the expression of C3 in an independent dataset (GSE99325). C3 was significantly overexpressed in the tubulointerstitium of patients with DN compared to normal controls (Figure 3).

### Relationship of C3 Expression in Renal Tubulointerstitium and Estimated Glomerular Filtration Rate

**FIGURE 4:**
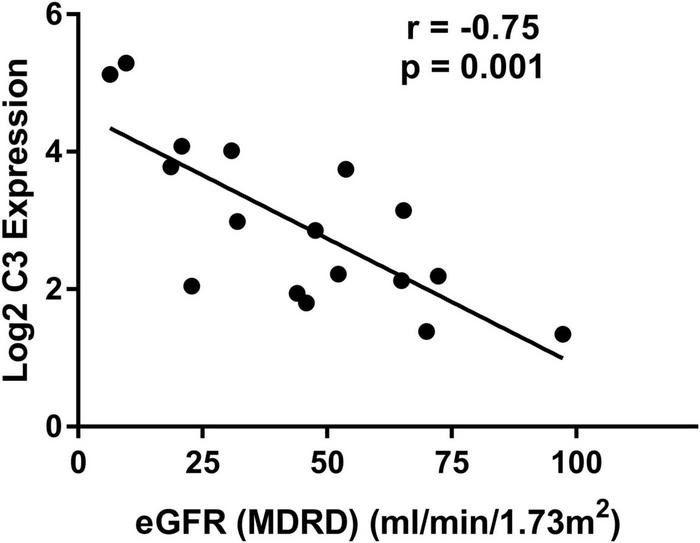
Correlation between mRNA expression of C3 in renal tubulointerstitium and eGFR in patients with diabetic nephropathy. eGFR, estimated glomerular filtration rate.

To validate the potential role of C3 in renal tubulointerstitial lesions in DN, a correlation analysis of C3 expression and eGFR was performed using Nephroseq v5 online tool. C3 mRNA level in renal tubulointerstitium was negatively correlated with eGFR in DN patients (*r* = −0.75; *p* = 0.001) (Figure 4), suggesting that high C3 expression accelerates kidney dysfunction.

### Association of C3 and C1q Deposits With Renal Outcome

A summary of the baseline data of 54 DN patients is presented in Table 3. Of the 54 patients with pure DN, the composite outcome of ESRD and a 50% reduction in the initial eGFR was noted in 29 cases. The mean follow-up duration was 22.1 ± 9.5 months. One patient reached ESRD within 6 months. Two patients died after progression to ESRD. Direct immunofluorescence microscopy revealed C3 deposits (C3 ≥ 1+), C1q deposits (C1q ≥ 1+), and C3 and C1q codeposits (C3 ≥ 1+ and C1q ≥ 1+) in 36 of 54 (66.7%), 26 of 54 (48.1%), and 23 of 54 (42.6%) patients, respectively. Deposition of C3 was found in the glomerular capillary walls, mesangium, Bowman’s capsule and tubular basement membrane. C1q was deposited in the glomerular capillary walls, mesangium and tubular basement membrane. There were significant differences in renal C3 deposits, C1q deposits, and C3 and C1q codeposits between the stable and progressive groups.

**TABLE 3:** Baseline data of 54 DN patients in the stable and progressive groups.

Variables	Stable (*n* = 25)	Progressive (*n* = 29)	*p*
Age (years)	52.6 ± 8.3	49.0 ± 9.6	0.2
Males, *n* (%)	18 (72%)	21 (72.4%)	0.9
Duration of diabetes (years)	9.6 ± 6.2	14.4 ± 5.3	0.003
UPE (g/24 h)	4.7 ± 4.1	6.6 ± 3.9	0.09
eGFR (ml/min/1.73 m^2^)	67.5 ± 23.8	44.6 ± 21.7	0.001
Serum C3 (mg/dl)	97.5 ± 18.9	88.6 ± 18.5	0.1
Serum C1q (mg/dl)	192.4 ± 59.3	192.9 ± 48.1	0.9
Renal C3 deposits, *n* (%)	12 (48%)	24 (82.8%)	0.007
Renal C1q deposits, *n* (%)	6 (24%)	20 (69%)	0.001
Renal C3 and C1q codeposits, *n* (%)	5 (20%)	18 (62.1%)	0.002
Using ACEI or ARB, *n* (%)	16 (60%)	18 (62.1%)	0.8

*eGFR, estimated glomerular filtration rate; UPE, urinary protein excretion; DN, diabetic nephropathy; ACEI, angiotensin-converting enzyme inhibitor; ARB, angiotensin II type I receptor blocker.*

Patients in the progressive group had a longer duration of diabetes, lower eGFR, and higher urinary protein excretion than those in the stable group. There were no significant differences in serum C3 or C1q levels between the two groups.

Table 4 shows the results of univariate and multivariate Cox regression analyses between baseline variables and the composite outcome in DN patients. Multivariate analysis of the follow-up data revealed significant associations between codeposits of renal C3 and C1q and the composite outcome (HR, 2.3; 95% CI, 1.01–5.2; *p* < 0.05). The baseline eGFR level also had the predictive value (HR, 0.94; 95% CI, 0.91–0.98; *p* < 0.05).

**TABLE 4:** Univariate and multivariate Cox regression analysis between baseline variables and definitive renal outcome.

Variables	Univariate analysis		Multivariate analysis		
	HR (95% CI)	*p*	β	HR (95% CI)	*p*
Age (years)	0.98 (0.9–1.0)	0.3			
Males, *n* (%)	1.8 (0.7–4.4)	0.2			
Duration of diabetes (years)	1.12 (1.04–1.21)	0.003	0.07	1.1 (0.9–1.2)	0.12
UPE (g/24 h)	1.1 (1.0–1.2)	0.05	0.08	1.1 (0.96–1.2)	0.17
eGFR (ml/min/1.73 m^2^)	0.94 (0.91–0.97)	<0.001	−0.06	0.94 (0.91–0.98)	<0.05
Serum C3 (mg/dl)	0.98 (0.96–1.003)	0.9			
Serum C1q (mg/dl)	1.003 (0.99–1.01)	0.4			
Renal C3 and C1q codeposits	2.6 (1.2–5.5)	0.02	0.8	2.3 (1.01–5.2)	<0.05
Using ACEI or ARB, *n* (%)	0.5 (0.3–1.2)	0.2			

*HR, hazard ratio; CI, confidence interval; eGFR, estimated glomerular filtration rate; UPE, urinary protein excretion; ACEI, angiotensin-converting enzyme inhibitor; ARB, angiotensin II type I receptor blocker.*

*β is the regression coefficient.*

### C3, C1q, MBL, C5b-9, and Factor B in Kidney Tissue

**FIGURE 5:**
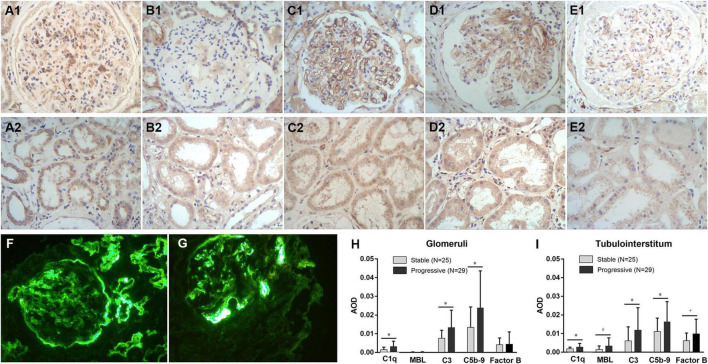
Immunohistochemical staining for C1q **(A1,A2)**, MBL **(B1,B2)**, C3 **(C1,C2)**, C5b-9 **(D1,D2)**, and factor B **(E1,E2)** expression in kidney tissues, the expression level of C1q, MBL, C3, C5b-9, and factor B in the stable and progressive groups **(H,I)**, and direct immunofluorescence microscopy for C3 **(F)** or C1q **(G)** deposits in kidney tissues. MBL, mannose-binding lectin; AOD, average optical density. *p < 0.05 versus stable group; ^#^*p* = 0.057 versus the stable group; ^†^*p* = 0.09 versus the stable group.

Figure 5 shows immunohistochemical staining results for C1q, MBL, C3, C5b-9, and factor B in the glomeruli and tubulointerstitium of patients with DN in the stable and progressive groups. Tubulointerstitial C1q, MBL, C3, and C5b-9 expression was significantly associated with adverse kidney outcomes (MBL, *p* = 0.057; C1q, C3, and C5b-9, *p* < 0.05, respectively). Expression of factor B was high in the progressive group, especially in renal tubulointerstitium, which was close to statistical significance (*p* = 0.09). High C1q, C3, and C5b-9 expression in glomeruli was also related to the progression of chronic kidney disease to kidney failure, although glomerular MBL expression was low irrespective of whether the patient had the composite outcome. This suggests that the classical complement pathway is activated in the glomeruli and tubulointerstitium during the progression of DN, whereas activation of the lectin complement pathway may be involved only in tubulointerstitial lesions. Alternative pathway may also play a role in the development of DN, but not necessarily the primary role.

## Discussion

This is the first study to use WGCNA to explore key genes in tubulointerstitial injury induced by diabetes. Nine co-expression modules were constructed from 12,075 genes from 38 human renal tubulointerstitial samples. Genes in the same module were considered functionally interrelated. The black module (2,779 genes) was the main one linked to tubulointerstitial injury in DN. In the black module, genes with the top 10 MCC values (hub genes) included ADAM10, CKAP4, C3, FBN1, TNC, SPP1, LAMC1, TIMP1, VCAN, and HSP90B1. Among them, a complement-related gene, C3 was further investigated through analysis of direct immunofluorescence and immunohistochemical data of 54 cases of DN.

Glomerular disease was formerly considered to be involved in the development and progression of DN (15). However, an increasing body of evidence supports an important role for tubulointerstitial injury, which is tightly correlated with DN progression (16, 17). The molecular mechanisms of linking tubulointerstitial lesions and glomerulopathy are poorly understood.

In this study, C3 was a key gene involved in tubulointerstitial injury in DN. Validation of an independent dataset from the GEO database showed that C3 was significantly overexpressed. Moreover, patients in the stable and progressive groups with C3 and C1q codeposits had poorer renal survival. These results implicate the classical complement pathway in DN.

The complement component C3 is at the core of all three pathways of complement activation (classical, lectin, and alternative) (18). The activation of C3 contributes to the production of C3b and, ultimately, generation of the C5b-9 complex, known as the membrane attack complex (MAC), which damages or activates target cells. Therefore, we examined the immunohistochemical expression of C1q (to measure the classical pathway), MBL (to measure the lectin pathway), factor B (to measure the alternative pathway), C5b-9, and C3 in tubulointerstitial and glomerular areas. Patients with high tubulointerstitial C1q, C3, C5b-9, and MBL levels were more likely to progress to kidney failure. Also, high expression of C1q, C3, and C5b-9 in glomeruli was linked to the progression of kidney failure, whereas glomerular MBL was rare and unrelated to renal survival. These findings indicate that the classical complement pathway is activated in glomeruli and the tubulointerstitium, whereas activation of the lectin pathway may be involved only in tubulointerstitial lesions. The expression of factor B did appear to be high in the glomeruli and tubulointerstitium of progressive DN although in a non-significant way. This does not necessarily indicate alternative pathway is not involved in diabetic kidney injury, but may not be the primary pathway of complement activation. Therefore, inhibition of the complement system may be a novel means to slow the progression of DN.

Diabetic nephropathy is not an “immune-mediated” kidney disease; instead, it is caused by prolonged exposure to a high serum glucose level. The effects of hyperglycemia on the vascular system are mediated by several factors, which ultimately result in the development and progression of DN. The factors that mediate the effects of hyperglycemia on the vasculature are typically divided into four categories: metabolic factors, hemodynamic factors, intracellular factors, and growth factors/cytokines (19). However, there is emerging evidence of a role for the complement system in the pathogenesis of DN (20–22).

In this study, although serum C3 level is not predictive of time to composite outcome, patients with C3 and C1q codeposits (*N* = 23) had a significant lower serum level of C3 (85.4 ± 15.3 mg/dl) compared to those without these deposits (*N* = 31; 97.2 ± 20.1 mg/dl) (data not shown). The decrease in circulating C3 level seen in patients with intrarenal C3 deposition might be the result of complement activation, in turn resulting in overconsumption of C3 and intrarenal deposition or excretion of urine proteins. In addition, the urinary protein excretion also failed to show a significant association with an increased risk of kidney function decline in multivariate Cox regression analysis. The level of proteinuria has typically significant influence on the progression of chronic kidney disease, but it decreases relatively when there is more global sclerosis in advanced DN. Based on pathology reports, patients with nephrotic-range proteinuria (*N* = 33; 8.1 ± 3.4 g/24 h) had similar proportions of global sclerosis (18.5% versus 19.1%) compared to those with non-nephrotic range proteinuria (*N* = 21; 1.9 ± 0.9 g/24 h). Combined with the proportion of global sclerosis and the number of follow-up events within 2 years, most of the patients involved were in the progression stage.

This study had several limitations. First, the role of complement in the development of DN was not determined. Second, whether the complement system is activated locally (in the kidneys) or systemically in DN is unclear. Finally, immunohistochemistry may show the deposition of complement-related compounds, not necessarily their local expression. Therefore, the role of complement in diabetic tubulointerstitial lesions warrants further investigation.

## Conclusion

In conclusion, local activation of the classical, lectin and alternative complement pathways in the kidneys may be involved in tubulointerstitial injury and disease progression in DN. Therefore, modulating of the complement system has therapeutic potential for DN.

## Data Availability Statement

The datasets presented in this study can be found in online repositories. The names of the repository/repositories and accession number(s) can be found in the article/Supplementary Material.

## Ethics Statement

The studies involving human participants were reviewed and approved by the Ethics Committee of China-Japan Friendship Hospital. The patients/participants provided their written informed consent to participate in this study.

## Author Contributions

SJ and WL contributed to conception and design of the study. SJ wrote the first draft of the manuscript. YJ organized the database and wrote sections of the manuscript. GZ and HG were responsible for histopathological preparation, staining, and results reading. LZ revised the final version. All authors contributed to the article and approved the submitted version.

## Conflict of Interest

The authors declare that the research was conducted in the absence of any commercial or financial relationships that could be construed as a potential conflict of interest.

## Publisher’s Note

All claims expressed in this article are solely those of the authors and do not necessarily represent those of their affiliated organizations, or those of the publisher, the editors and the reviewers. Any product that may be evaluated in this article, or claim that may be made by its manufacturer, is not guaranteed or endorsed by the publisher.

## Footnotes

1
http://www.ncbi.nlm.nih.gov/geo/

2
http://david.ncifcrf.gov/summary.jsp

3
http://www.string-db.org

4
http://cytoscape.org/

5
http://www.R-project.org/

6
https://www.nephroseq.org/
