# Supplementary material for: Activation of Complement Pathways in Kidney Tissue May Mediate Tubulointerstitial Injury in Diabetic Nephropathy
Source: Front Med (Lausanne). 2022 Apr 11;9:845679. doi: 10.3389/fmed.2022.845679 (PMC9037626; doi:10.3389/fmed.2022.845679)
Supplement: Supplementary file 1 [file Data_Sheet_1.docx]

Supplementary Material


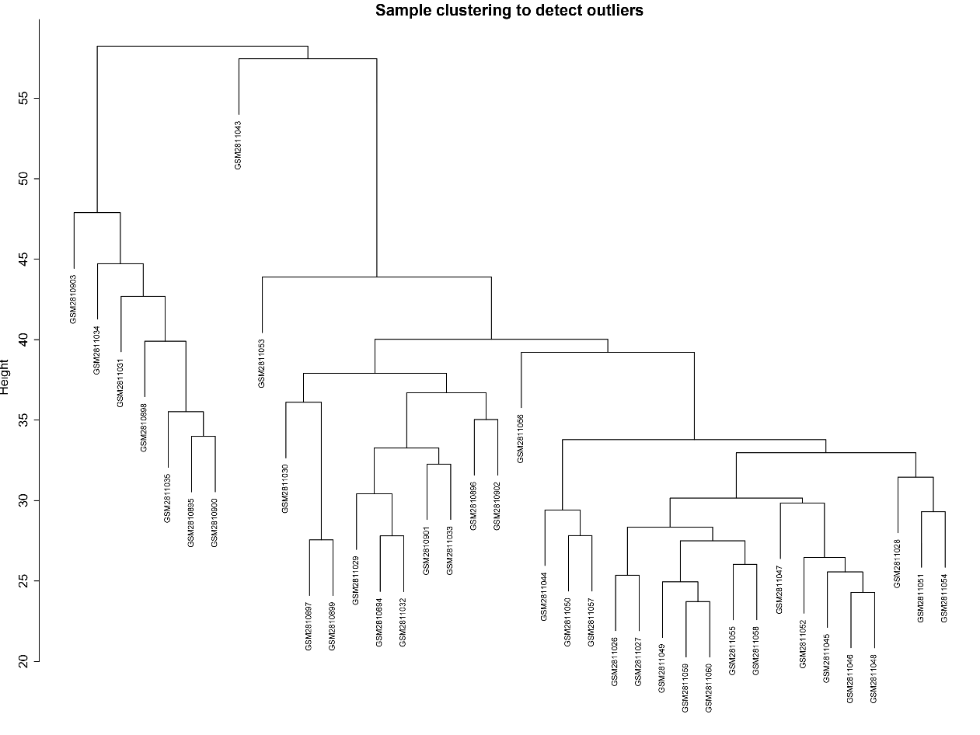


**Supplementary Figure 1.** Sample clustering to detect outliers. All the samples were in the clusters, and all the samples have passed the cuts.


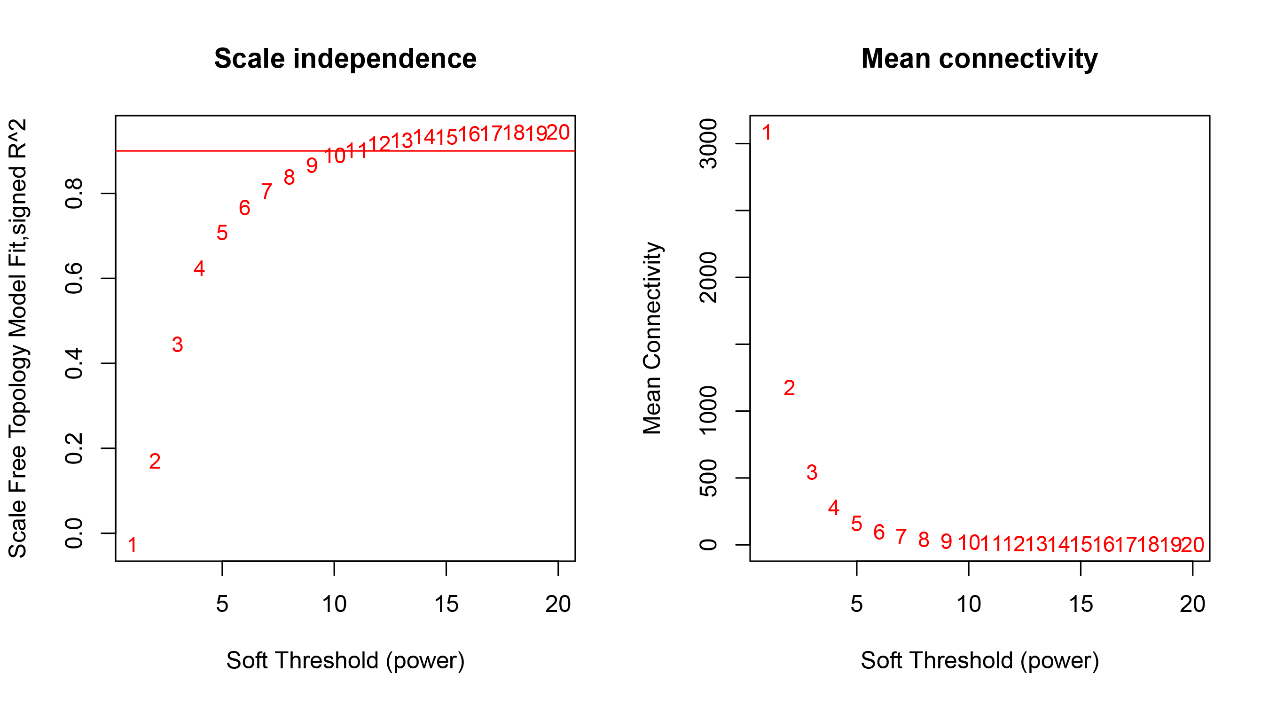


**Supplementary Figure 2.** Analysis of scale-free fit index for various soft-thresholding powers (Left) and analysis of the mean connectivity for various soft-thresholding powers (Right).


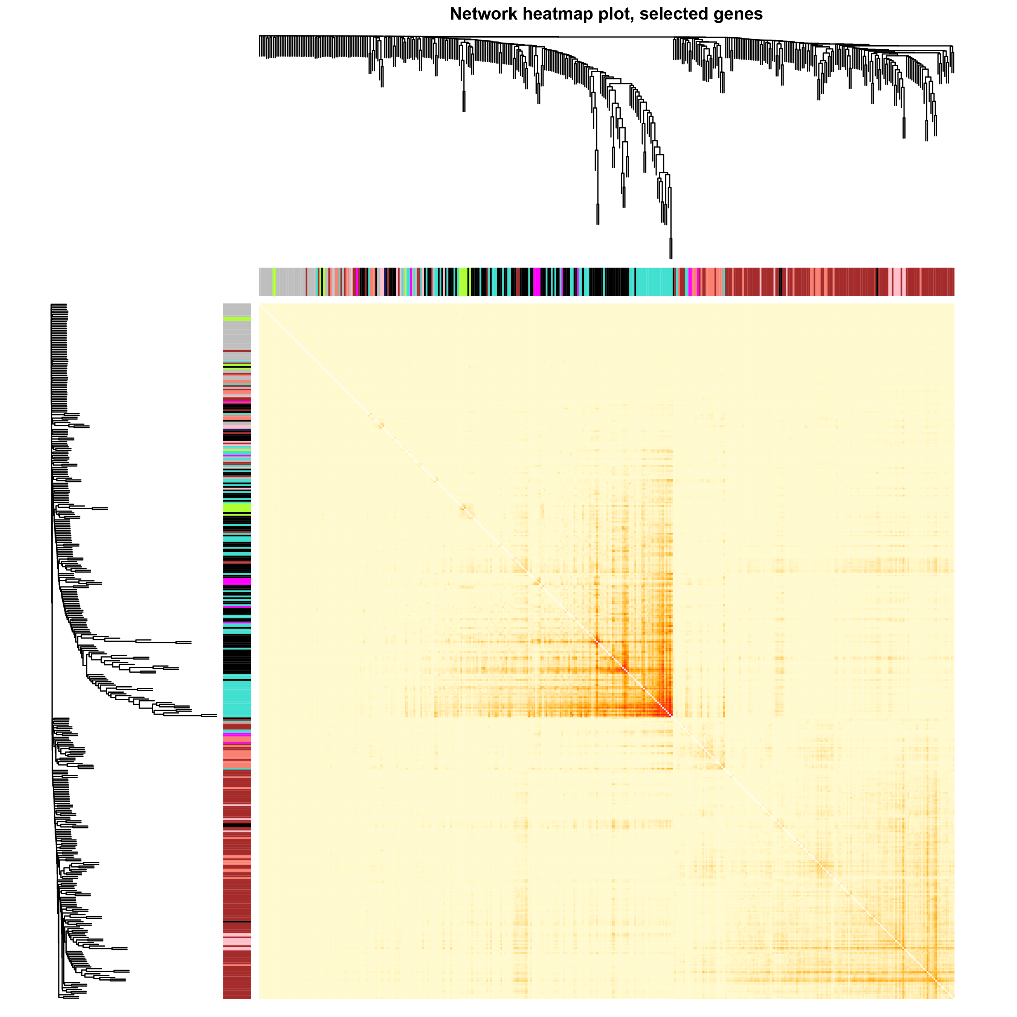


**Supplementary Figure 3.** Heatmap plot of the 400 selected genes. The colors from light to deep red represent a low to high overlap. The gene dendrogram and module assignment are shown along the left and the top of the figure.


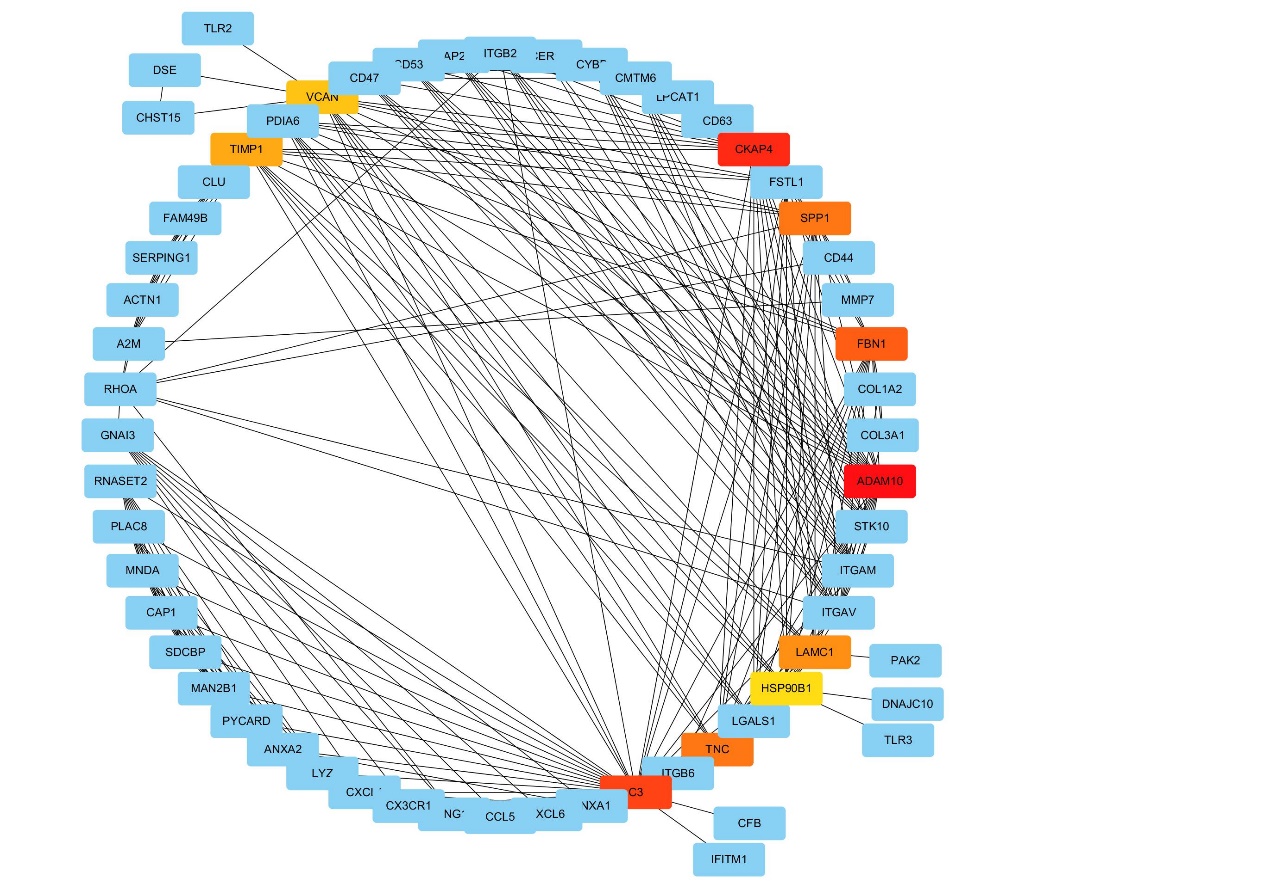


**Supplementary Figure 4.** Hub genes from the black module based on Cytoscape. The export network to Cytoscape package is used to obtain a network in edge and node gene list files based on the WGCNA method. This figure displays the gene network of rank 10. The darker the color, the higher the ranking.
